# A Case of Bladder Rupture Initially Misdiagnosed as Ascites Due to Alcoholic Liver Disease

**DOI:** 10.7759/cureus.101073

**Published:** 2026-01-08

**Authors:** Toshikazu Ozeki, Shun Ito, Takuya Sugiura, Yuki Yokoe, Kaoru Yasuda

**Affiliations:** 1 Nephrology, Japanese Red Cross Aichi Medical Center Nagoya Daiichi Hospital, Nagoya, JPN; 2 Nephrology, Chubu Rosai Hospital, Nagoya, JPN; 3 Nephrology, Kasugai Municipal Hospital, Kasugai, JPN; 4 Nephrology, Nagoya University, Nagoya, JPN

**Keywords:** acute kidney injury, ascites, bladder rupture, postrenal acute kidney injury, pseudo-renal failure

## Abstract

Bladder rupture is a rare condition, typically associated with trauma but occasionally occurring spontaneously. Non-traumatic bladder rupture is particularly challenging to diagnose due to its non-specific symptoms. This case report presents a bladder rupture initially misdiagnosed as ascites due to alcoholic liver disease. A 59-year-old man presented with progressive abdominal distension without abdominal pain or urinary symptoms. His medical history included diabetes, asthma, dyslipidemia, and chronic alcohol consumption. Laboratory tests showed renal impairment but no significant liver dysfunction. Abdominal CT revealed ascitic fluid, leading to a diagnosis of alcoholic liver disease. He was hospitalized, and conservative treatment was started, but his renal function deteriorated. On the fifth day, however, after urinary catheterization, 10 L of urine were drained within six hours, resulting in a rapid decrease in serum creatinine levels. Retrospective review of imaging revealed a rupture at the bladder apex, likely caused by chronic bladder overdistension due to alcohol use and diabetic autonomic dysfunction. The patient recovered with conservative management, and a follow-up cystogram confirmed bladder healing. This case highlights the diagnostic challenge of non-traumatic bladder rupture, especially in the absence of typical symptoms. Clinicians should consider bladder rupture in patients presenting with unexplained ascites, renal impairment, and an unusual diuretic response to catheterization.

## Introduction

Bladder rupture is a rare clinical entity, typically associated with trauma but occasionally occurring spontaneously. The diagnosis of non-traumatic bladder rupture is particularly challenging due to its non-specific clinical presentation [1]. Here, we report a case of spontaneous bladder rupture that was initially misdiagnosed as ascites secondary to alcoholic liver disease, owing to the absence of characteristic urinary symptoms.

## Case presentation

A 59-year-old man presented with progressive abdominal distension in the absence of abdominal pain or urinary symptoms. His medical history included type 2 diabetes mellitus, insomnia, bronchial asthma, dyslipidemia, and hyperuricemia. His regular medications included allopurinol, empagliflozin, linagliptin, rosuvastatin, brotizolam, and a salmeterol/fluticasone inhaler. He reported daily alcohol consumption of approximately 700 mL of beer and 1,000 mL of strong canned chuhai. The patient did not report clear antecedent lower urinary tract symptoms such as a weak stream, hesitancy, or sensation of incomplete emptying before admission.

On admission, his height was 165 cm, and his body weight was 76 kg. Vital signs were as follows: blood pressure, 143/105 mmHg; heart rate, 96 beats per minute; temperature, 36.6°C; respiratory rate, 16 breaths per minute; and oxygen saturation, 96% on room air. Initial laboratory investigations revealed the findings summarized in Table 1, including elevated serum creatinine (3.00 mg/dL), hyperglycemia (blood glucose, 322 mg/dL; HbA1c, 8.3%), and microscopic hematuria without proteinuria on urinalysis.

**Table 1:** Laboratory findings. AST = aspartate aminotransferase; ALT = alanine aminotransferase; BUN = blood urea nitrogen; Cr = creatinine; PT-INR = prothrombin time–international normalized ratio; APTT = activated partial thromboplastin time; HbA1c = glycated hemoglobin

Parameter	Result	Reference range	Unit
Serum albumin	4.7	4.1–5.1	g/dL
AST	27	13–30	U/L
ALT	43	10–42	U/L
Total bilirubin	1.3	0.4–1.5	mg/dL
BUN	37	8–20	mg/dL
Cr	3	0.65–1.07	mg/dL
White blood cell count	11,300	3,300–8,600	/µL
Hemoglobin	17.3	13.7–16.8	g/dL
Platelet count	20.9	15.8–34.8	×10⁴/µL
PT-INR	0.99	0.90–1.15	-
APTT	33.4	25.0–37.0	seconds
HbA1c	8.3	4.0–5.6	%
Blood glucose	322	<140	mg/dL
Proteinuria	−	−	-
Hematuria	+	−	-

**Figure 1:**
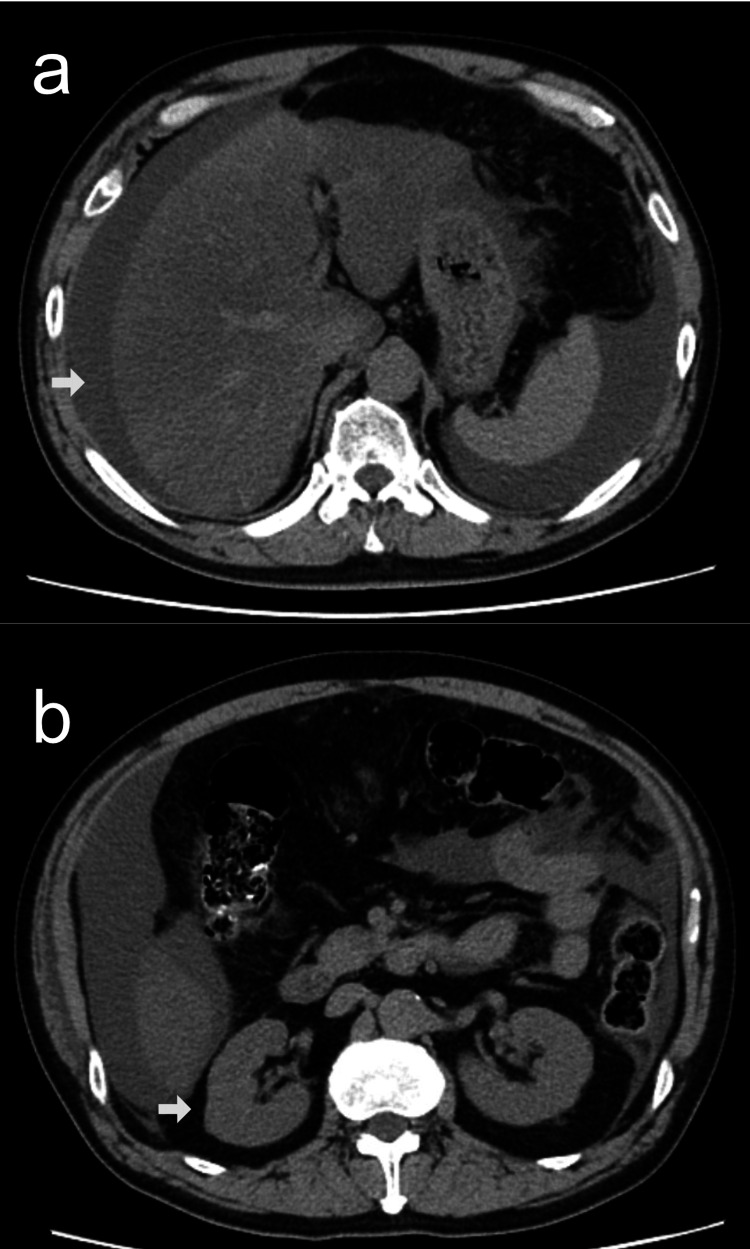
Abdominal axial CT scan on admission. Ascites and liver atrophy (a), with no apparent abnormalities in the kidneys (b).

Although laboratory data did not indicate significant hepatic dysfunction, abdominal CT demonstrated the presence of ascites and hepatic parenchymal irregularity (Figure 1a). In the absence of an alternative etiology, the ascites was attributed to presumed alcoholic liver disease. Renal morphology appeared normal on imaging (Figure 1b), and hepatorenal syndrome was considered a potential cause of renal impairment. The patient was admitted and initiated on diuretic therapy.

During the initial days of hospitalization, his urine output ranged between 500 and 1,000 mL per day. However, his serum creatinine rose to 6.68 mg/dL by day five. To better monitor urine output, a urinary catheter was placed, resulting in the drainage of 10 L of urine within six hours. Notably, no intravenous fluids were administered. By the following day, his serum creatinine had markedly decreased to 0.81 mg/dL, and his body weight had dropped from 79.4 kg to 70.3 kg.

**Figure 2:**
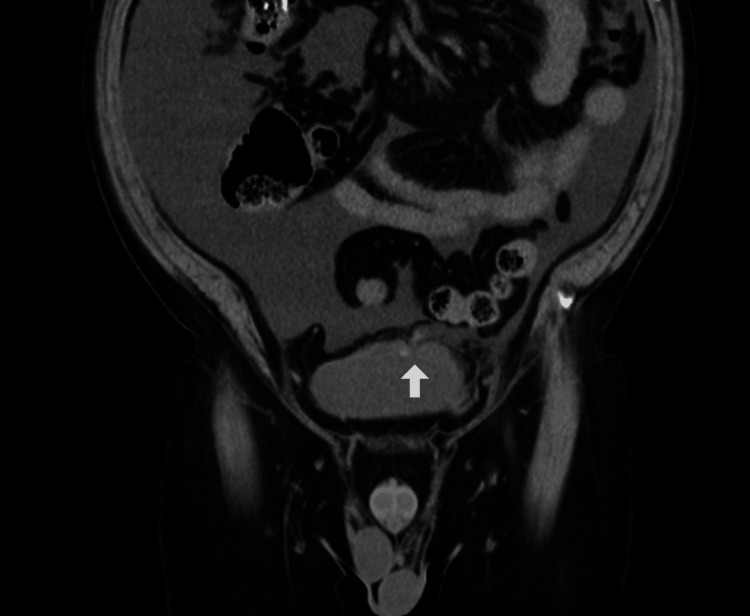
Abdominal coronal CT scan on admission. The image reveals a communication between the bladder apex and the peritoneal cavity (arrow).

Although post-renal acute kidney injury was initially considered, follow-up CT imaging showed complete resolution of the ascites. Retrospective review of the admission CT scan identified a rupture at the bladder apex (Figure 2). Given the patient’s history of heavy alcohol use and diabetic autonomic neuropathy, chronic bladder overdistension was presumed to be the underlying cause of bladder rupture.

The patient was managed conservatively with an indwelling urinary catheter. After 15 days, a cystogram confirmed the absence of contrast extravasation, and the catheter was removed without evidence of residual urinary retention. After discharge, the patient was scheduled for outpatient urology follow-up to evaluate for underlying voiding dysfunction and prevent recurrence. Planned assessments included uroflowmetry and post-void residual measurement, with further evaluation such as cystoscopy as clinically indicated. The patient was counseled to avoid heavy alcohol intake and seek prompt medical attention if abdominal distension, reduced urine output, or urinary retention recur.

## Discussion

Bladder rupture is most commonly associated with trauma; however, spontaneous cases have been documented. Recognized risk factors for non-traumatic bladder rupture include neurogenic bladder due to diabetes mellitus, transurethral resection of bladder tumors, pelvic irradiation, and chronic excessive alcohol intake, which may lead to bladder overdistension [1,2]. In the present case, a combination of longstanding alcohol consumption and diabetic autonomic dysfunction likely predisposed the patient to bladder rupture.

A key diagnostic challenge in this case was the absence of typical clinical features. Approximately 80% of bladder rupture cases present with abdominal pain, and a similar proportion report urinary symptoms [1,3]. However, our patient exhibited neither, and his preserved urine output during the early phase of hospitalization further delayed the recognition of the underlying pathology. Despite normal hepatic function, the presence of ascites and a history of alcohol use led to an initial misdiagnosis of alcoholic liver disease. Although alcoholic liver disease and hepatorenal syndrome were initially considered due to the presence of ascites, hepatic synthetic function was preserved, and there was no evidence of advanced cirrhosis. Importantly, the rapid normalization of urine output and serum creatinine after bladder drainage was inconsistent with hepatorenal syndrome and strongly supported urinary ascites with pseudo-renal failure.

Although post-obstructive diuresis can result in urine output exceeding 10 L per day [4,5], the rapid drainage observed in our patient suggested preexisting intraperitoneal accumulation rather than increased production. While renal function typically recovers gradually over several days following relief of obstruction [5], this patient experienced an immediate and dramatic improvement in renal parameters, which is more consistent with so-called pseudo-renal failure. This phenomenon results from reabsorption of creatinine across the peritoneal membrane, and rapid normalization of serum creatinine levels has been reported following drainage of urine from the abdominal cavity [6,7].

Importantly, the absence of hydronephrosis on initial CT imaging made post-renal acute kidney injury less likely. Although hydronephrosis may be absent in the early stages of obstruction or in cases with low urine output [8], such scenarios are relatively uncommon. In this context, the abrupt resolution of ascites following catheterization strongly suggested the presence of a urinary-peritoneal fistula.

Notably, diagnostic paracentesis was not performed during the initial assessment of ascites, which, in hindsight, should have been undertaken. Ascitic fluid analysis allows for the differentiation between transudative and exudative ascites, aiding in distinguishing cirrhosis from other etiologies such as malignancy, tuberculosis, or gastrointestinal perforation. Previous studies have demonstrated that creatinine concentrations in ascitic fluid rise significantly in the presence of urinary leakage into the peritoneal cavity, and an elevated ascitic fluid-to-serum creatinine ratio serves as a valuable diagnostic clue for urinary ascites [9]. Therefore, earlier performance of paracentesis might have facilitated a more timely and accurate diagnosis in this case.

## Conclusions

This case underscores the diagnostic difficulty of non-traumatic bladder rupture, particularly in the absence of characteristic signs and symptoms. Clinicians should maintain a high index of suspicion for bladder rupture in patients presenting with unexplained ascites, renal dysfunction, and an atypical diuretic response following urinary catheterization.
